# Analysis of clinicopathological features and prognosis of mesenteric versus anti-mesenteric rectal cancer: a single-center retrospective cohort study

**DOI:** 10.3389/fonc.2026.1801287

**Published:** 2026-07-10

**Authors:** Dalei Hao, Longzhan Dong, Xiangpeng Xi, Yulin Liu, Yongbo Zhang, Kang Xu, Jingbo Chen

**Affiliations:** 1 School of Clinical Medicine, Shandong Second Medical University, Weifang, China; 2 Department of General Surgery, The First Affiliated Hospital of Shandong First Medical University& Shandong Provincial Qianfoshan Hospital, Jinan, China

**Keywords:** anti-mesenteric side, axial position, local recurrence, magnetic resonance imaging, mesenteric side, prognosis, rectal cancer

## Abstract

**Background:**

The mesorectal and anti-mesorectal sides of the rectum differ considerably in embryonic origin, blood supply, lymphatic distribution, and anatomical relations. Tumor location (mesenteric vs. anti-mesenteric) may therefore influence tumor biology and clinical outcomes. This study aimed to evaluate the impact of axial tumor location on clinicopathological features and prognosis in rectal cancer using preoperative high-resolution pelvic MRI.

**Methods:**

We retrospectively reviewed 380 rectal cancer patients who underwent radical resection between January 2017 and July 2023. Based on preoperative MRI axial images, tumors were classified by their deepest point of invasion relative to the rectal lumen: the mesenteric side group (3–9 o’clock, posterior/posterolateral walls, n=213) and the anti-mesenteric side group (9–3 o’clock, anterior/anterolateral walls, n=167). Demographic, clinicopathological, surgical, and survival data were compared between groups.

**Results:**

Among the 380 patients, 213 were assigned to the mesenteric side tumor group and 167 to the anti-mesenteric side tumor group. Baseline characteristics, including age, gender, BMI, TNM stage, MRF status, EMVI, and vascular/nerve invasion, did not differ significantly between two groups (all P > 0.05). With a median follow-up of 56 months, the 3-year local recurrence-free survival (LRFS) rate was significantly lower in the anti-mesenteric group than in the mesenteric group (91.6% vs. 97.1%, P = 0.029). No significant differences were observed in 3-year disease-free survival (83.2% vs. 82.1%, P = 0.832) or overall survival (85.6% vs. 81.7%, P = 0.501) between the two groups. Multivariable Cox regression analysis identified age (HR = 1.043, P = 0.002), surgical procedure (APR vs. LAR, HR = 1.967, P = 0.022), pathological T stage (HR = 2.800, P = 0.023), and pathological N stage (HR = 3.683, P < 0.001) as independent prognostic factors for overall survival. Pathological N stage was the sole independent predictor for disease-free survival (HR = 3.088, P < 0.001). Although axial location was not an independent predictor of overall or disease-free survival (P > 0.05), it was significantly associated with LRFS (anti-mesenteric vs. mesenteric: HR = 2.684, 95% CI: 1.126–6.398, P = 0.026) along with pathological N stage (HR = 3.960, 95% CI: 1.316–11.919, P = 0.014).These findings suggest that anti-mesenteric tumor location is an independent predictor of increased local recurrence risk, providing valuable information for surgical planning and postoperative surveillance beyond conventional staging.

**Conclusion:**

Anti-mesenteric rectal tumors are associated with a higher risk of local recurrence, likely due to complex local anatomy and surgical challenges. While pathological N stage, pathological T stage, age, and surgical procedure remain primary independent prognostic factors for survival outcomes, preoperative MRI assessment of axial location provides valuable supplemental information that may help stratify local recurrence risk, refine surgical planning, and optimize postoperative monitoring.

## 1 Introduction

Rectal cancer remains a leading cause of cancer-related morbidity and mortality worldwide (1). Total mesorectal excision (TME) has markedly improved local control, yet patient outcomes vary considerably (2, 3). While the impact of longitudinal tumor location (upper, middle, lower rectum) on treatment and prognosis is well established (4, 5), the influence of circumferential (axial) location is less understood.

Anatomically, the rectal wall exhibits regional heterogeneity (6, 7). The mesorectal side (posterior/posterolateral walls) derives from the dorsal mesentery, is enveloped by the mesorectum, and offers a well-defined dissection plane for TME (8). In contrast, the anti-mesenteric side (anterior/anterolateral walls) lacks a clear fascial boundary and is adjacent to structures such as the prostate, seminal vesicles, or posterior vaginal wall, which may complicate resection and increase recurrence risk (9). Preliminary evidence suggests that tumors on the anti-mesenteric side may be associated with poorer local control (10–13), but robust prospective data are lacking, particularly based on standardized preoperative imaging.

In this study, we used preoperative high-resolution pelvic MRI to classify rectal tumors by axial location (mesenteric vs. anti-mesenteric side) and compared clinicopathological characteristics and survival outcomes between the two groups.

## 2 Materials and methods

### 2.1 Patient selection

**Figure 1:**
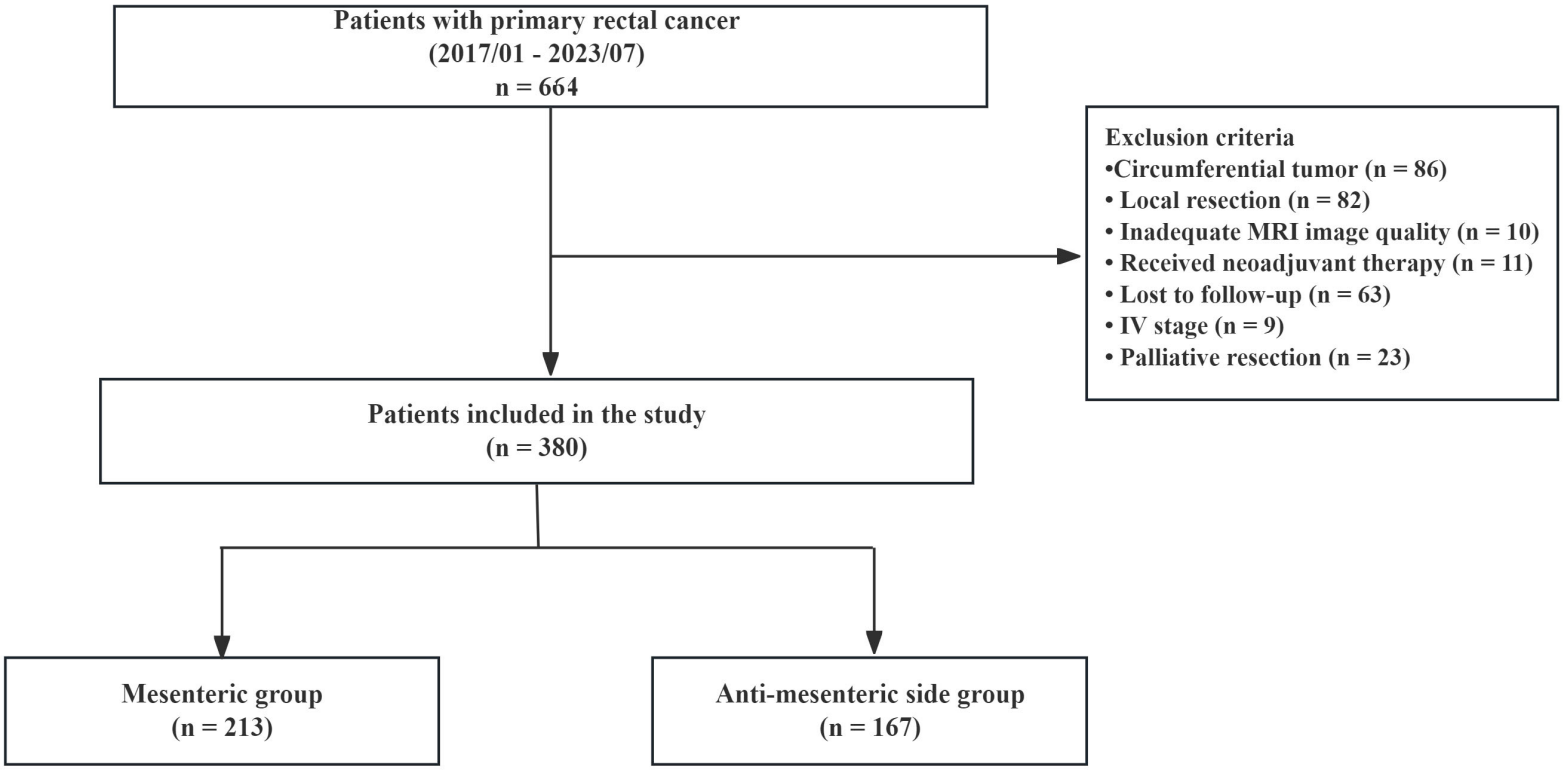
Study flow diagram for patient inclusion.

This single-center retrospective cohort study included patients who underwent radical surgery for primary rectal adenocarcinoma between January 2017 and July 2023. Of the 664 initially screened patients, 380 met the inclusion criteria: (1) pathologically confirmed primary rectal adenocarcinoma; (2) curative-intent radical resection; (3) available high-quality preoperative pelvic MRI for tumor localization; (4) complete clinicopathological and follow-up data. Exclusion criteria were: (1) palliative resection; (2) local excision (TEM); (3) inadequate MRI image quality (n = 10); (4) received any form of neoadjuvant therapy prior to surgery, resulting in an absence of baseline MRI for natural anatomical evaluation (n = 11); (5) circumferential tumor growth >75%; (6) incomplete data or loss to follow-up; (7) stage IV disease. By focusing exclusively on an upfront surgery cohort, we aimed to eliminate the confounding effects of radiation-induced tissue fibrosis and tumor downstaging on natural anatomical planes. It is important to note that our cohort includes a proportion of patients with clinically staged T3 or node-positive (cN+) disease who underwent upfront surgery. In our real-world clinical practice during the study period, these deviations from standard neoadjuvant chemoradiotherapy (nCRT) guidelines were carefully evaluated by our multidisciplinary team (MDT). Upfront surgery was selectively performed primarily due to explicit patient refusal of nCRT (often stemming from concerns regarding radiation-induced toxicity, prolonged treatment duration, or socioeconomic factors), or when patients were deemed medically unfit for rigorous neoadjuvant regimens. The study flowchart is shown in **Figure 1**. Ethical approval was obtained (YXLL-KY-2025(227)).

### 2.2 Data collection and definition

**Figure 2:**
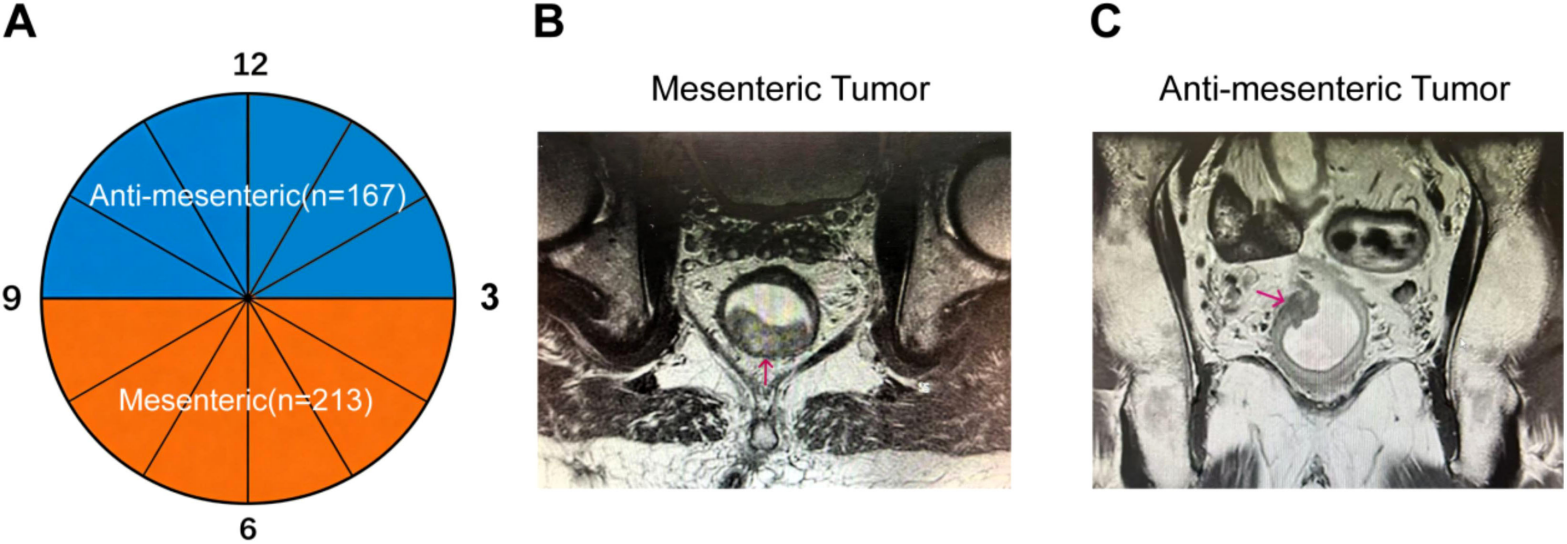
**(A)** Rectal tumor location in patients placed in the lithotomy position is categorized as follows: mesenteric tumors are situated between the 3 and 9 o’clock positions, while anti-mesenteric tumors are located between the 9 and 3 o’clock positions. Corresponding axial contrast-enhanced MRI images demonstrate the deepest extent of tumor invasion in the mesenteric **(B)** and anti-mesenteric **(C)** regions of the rectum.

Clinical, pathological, and imaging data were retrieved from a prospectively maintained database. Two experienced radiologists, blinded to outcomes, independently reviewed preoperative MRI to determine tumor location, size, relation to the mesorectal fascia (MRF), clinical TNM stage, and extramural vascular invasion (EMVI). Axial location was classified on T2-weighted images using the rectal lumen center as a reference. Tumors with deepest invasion between 3 and 9 o’clock (posterior/posterolateral) were classified as mesenteric side, and those between 9 and 3 o’clock (anterior/anterolateral) as anti-mesenteric side (**Figure 2**). TNM staging followed the AJCC 8th edition (13).

### 2.3 Surgical treatment and adjuvant therapy

All surgeries were performed by colorectal surgeons (over 100 cases per year), based on total mesorectal excision. Positive circumferential resection margin was defined as tumor ≤ 1 mm from the resection margin. Low anterior resection and abdominoperineal resection were chosen depending on tumor location. Surgeries were performed via open or minimally invasive (conventional laparoscopic) approaches. In our center, laparoscopic total mesorectal excision is the preferred standard approach for the majority of patients. Robotic-assisted surgeries were not performed in this cohort. Open surgery was reserved for specific scenarios based on rigorous surgeon evaluation, such as a history of extensive abdominal surgeries with suspected severe adhesions, huge tumor size causing acute obstruction, or severe cardiopulmonary comorbidities contraindicating pneumoperitoneum. Postoperative adjuvant chemotherapy followed National Comprehensive Cancer Network guidelines tailored to patient condition (14). The main regimens were FOLFOX, XELOX or single-agent fluoropyrimidine therapy.

### 2.4 Follow-up and outcome indicators

Regular follow-up after surgery was carried out by outpatient reexamines, telephone calls and electronic medical records. Physical exams, serum tumor markers, CT scans of chest, abdomen, pelvis, colon, colon, etc. Follow-up was carried out every 3 months during first year after surgery, every 6 months from second year to third year and annually thereafter. Local recurrence-free survival (LRFS) was defined as the time from surgery to the first confirmed local (pelvic) recurrence. Disease-free survival (DFS) was defined as the time from surgery to any recurrence, distant metastasis, or death from any cause. Overall survival (OS) was defined as the time from surgery to death from any cause. Recurrence was confirmed by imaging or pathological examination.

### 2.5 Statistical analysis

Data were analyzed using IBM SPSS Statistics version 25.0. Continuous variables with normal distributions were presented as mean ± standard deviation (SD), and comparisons between groups were performed using the independent-samples t-test. Continuous variables with non-normal distributions were expressed as median (interquartile range, IQR) and compared using the Mann-Whitney U test. Categorical variables were presented as frequencies (percentages), and group comparisons were evaluated using the chi-square test or Fisher’s exact test, as appropriate. Survival curves were generated using the Kaplan-Meier method, and differences between groups were evaluated using the log-rank test. Univariable and multivariable Cox proportional hazards regression models were used to identify independent prognostic factors affecting survival. Variables with P < 0.10 in the univariable analysis, or those considered clinically relevant, were included in the multivariable analysis. All statistical tests were two-sided, and a p-value < 0.05 was considered statistically significant. Furthermore, to evaluate the potential confounding impact of the baseline imbalance in surgical procedures (APR vs. LAR) between the mesenteric and anti-mesenteric groups, a sensitivity analysis for local recurrence-free survival (LRFS) was performed. Specifically, an expanded multivariable Cox proportional hazards model was constructed by explicitly forcing the surgical procedure into the regression model as an additional covariate, alongside the pre-established variables (axial tumor location, pathological N stage, and perineural invasion).

## 3 Results

### 3.1 Baseline characteristics of patients

A total of 380 patients who underwent radical resection for rectal cancer were included, with 237 (62.37%) males and 143 (37.63%) females, and a mean age of 62.47 ± 10.83 years. Based on preoperative axial MRI, tumors were classified into the mesenteric side group (n = 213, 56.05%) and the anti-mesenteric side group (n = 167, 43.95%). No statistically significant differences were observed between the two groups in age (P = 0.486), sex (P = 0.435), BMI (P = 0.735), MRI-assessed MRF status (P = 0.811), EMVI (P = 0.240), clinical T stage (P = 0.262), clinical N stage (P = 0.790), clinical TNM stage (P = 0.297), or longitudinal tumor location (P = 0.971), indicating comparable baseline characteristics (**Table 1**).

**Table 1:** Demographics and baseline clinical characteristics.

Variables	n (%) or mean ± SD			
	Total (n = 380)	Mesenteric (n = 213)	Anti-mesenteric (n = 167)	P
Age	62.47 ± 10.83	62.17 ± 10.79	62.85 ± 10.90	0.486
Gender				0.435
Female	143 (37.63)	76 (35.68)	67 (40.12)	
Male	237 (62.37)	137 (64.32)	100 (59.88)	
BMI (kg/m2)	24.10 ± 3.50	24.14 ± 3.53	24.06 ± 3.45	0.735
MRF				0.811
Positive (+)	36 (9.47)	19 (8.92)	17 (10.18)	
Negative (-)	344 (90.53)	194 (91.08)	150 (89.82)	
EMVI				0.240
Positive (+)	193 (50.79)	102 (47.89)	91 (54.49)	
Negative (-)	187 (49.21)	111 (52.11)	76 (45.51)	
cT stage				0.262
T1	7 (1.84)	5 (2.35)	2 (1.20)	
T2	109 (28.68)	55 (25.82)	54 (32.34)	
T3	162 (42.63)	89 (41.78)	73 (43.71)	
T4	102 (26.84)	64 (30.05)	38 (22.75)	
cN stage				0.790
N0	182 (47.9)	101 (47.4)	81 (48.5)	
N1	145 (38.2)	80 (37.6)	65 (38.9)	
N2	53 (13.9)	32 (15.0)	21 (12.6)	
cTNM stage				0.297
I	94 (24.74)	47 (22.07)	47 (28.14)	
II	88 (23.16)	54 (25.35)	34 (20.36)	
III	198 (52.11)	112 (52.58)	86 (51.50)	
Longitudinal tumor location				0.971
Above peritoneal reflection	135 (35.53)	75 (35.21)	60 (35.93)	
Below peritoneal reflection	245 (64.47)	138 (64.79)	107 (64.07)	

BMI, Body Mass Index; MRF, Mesorectal Fascia; EMVI, Extramural Vascular Invasion.

### 3.2 Perioperative and pathological outcomes

All patients successfully underwent radical surgery, including low anterior resection (LAR) in 318 cases (83.68%) and abdominoperineal resection (APR) in 62 cases (16.32%). No significant differences were found in operative time or estimated blood loss between the two groups. Notably, the proportion of APR was significantly higher in the mesenteric group than in the anti-mesenteric group (20.7% vs. 10.8%, P = 0.014). Pathological outcomes, including tumor differentiation, pT stage, pN stage, pCRM positivity rate, vascular invasion, and perineural invasion, did not differ significantly between the two groups (all P > 0.05), as summarized in **Table 2**.

**Table 2:** Perioperative and pathological results.

Variables	Total (n = 380)	Mesenteric (n = 213)	Anti-mesenteric (n = 167)	P
Operative time (min)	190.15 ± 43.43	192.85 ± 44.17	186.71 ± 42.36	0.172
Blood loss (ml)	50.65 ± 36.09	51.38 ± 29.98	49.72 ± 42.70	0.656
Surgical procedure, n (%)				0.014
APR	62 (16.32)	44 (20.66)	18 (10.78)	
LAR	318 (83.68)	169 (79.34)	149 (89.22)	
Surgical approach, n (%)				0.092
Laparoscopic	359 (94.47)	197 (92.49)	162 (97.01)	
Open	21 (5.53)	16 (7.51)	5 (2.99)	
Vascular invasion, n (%)				0.272
Positive (+)	170 (44.74)	103 (48.36)	67 (40.12)	
Negative (-)	201 (52.89)	105 (49.30)	96 (57.49)	
Unknown	9 (2.37)	5 (2.35)	4 (2.40)	
Perineural invasion, n (%)				0.760
Positive (+)	170 (44.74)	92 (43.19)	78 (46.71)	
Negative (-)	202 (53.15)	116 (54.46)	86 (51.50)	
Unknown	8 (2.11)	5 (2.35)	3 (1.80)	
Differentiation, n (%)				0.988
Well	7 (1.8)	4 (1.9)	3 (1.8)	
Moderate	315 (82.9)	176 (82.6)	139 (83.2)	
Poor	58 (15.3)	33 (15.5)	25 (15.0)	
Tumor size (cm), n (%)				0.996
< 5	240 (63.2)	134 (62.9)	106 (63.5)	
≥ 5	140 (36.8)	79 (37.1)	61 (36.5)	
pT stage, n (%)				0.418
T1	11 (2.89)	6 (2.82)	5 (2.99)	
T2	100 (26.32)	49 (23.00)	51 (30.54)	
T3	217 (57.11)	127 (59.62)	90 (53.89)	
T4	52 (13.68)	31 (14.55)	21 (12.57)	
pN stage, n (%)				0.396
N0	209 (55.00)	112 (52.58)	97 (58.08)	
N1	97 (25.53)	60 (28.17)	37 (22.16)	
N2	74 (19.47)	41 (19.25)	33 (19.76)	
pTNM stage, n (%)				0.373
I	90 (23.68)	45 (21.13)	45 (26.95)	
II	119 (31.32)	67 (31.46)	52 (31.14)	
III	171 (45.00)	101 (47.42)	70 (41.92)	
pCRM, n (%)				0.832
Positive (+)	3 (0.8)	2 (0.9)	1 (0.6)	
Negative (-)	377 (99.2)	211 (99.1)	166 (99.4)	

APR, Abdominoperineal resection; LAR, Low anterior resection; TNM, Tumor, Node, Metastasis; pCRM, Pathological circumferential resection margin.

### 3.3 Survival and recurrence outcomes

The median follow-up duration was 56 months (range: 6–106 months). During follow-up, 66 patients (17.4%) developed recurrence or metastasis. Specifically, a total of 23 patients experienced local pelvic recurrence (comprising 15 isolated local recurrences and 8 combined local and distant recurrences), resulting in an overall local recurrence rate of 6.1% (23/380). Additionally, 43 patients (11.3%) presented with distant metastasis alone.

**Figure 3:**
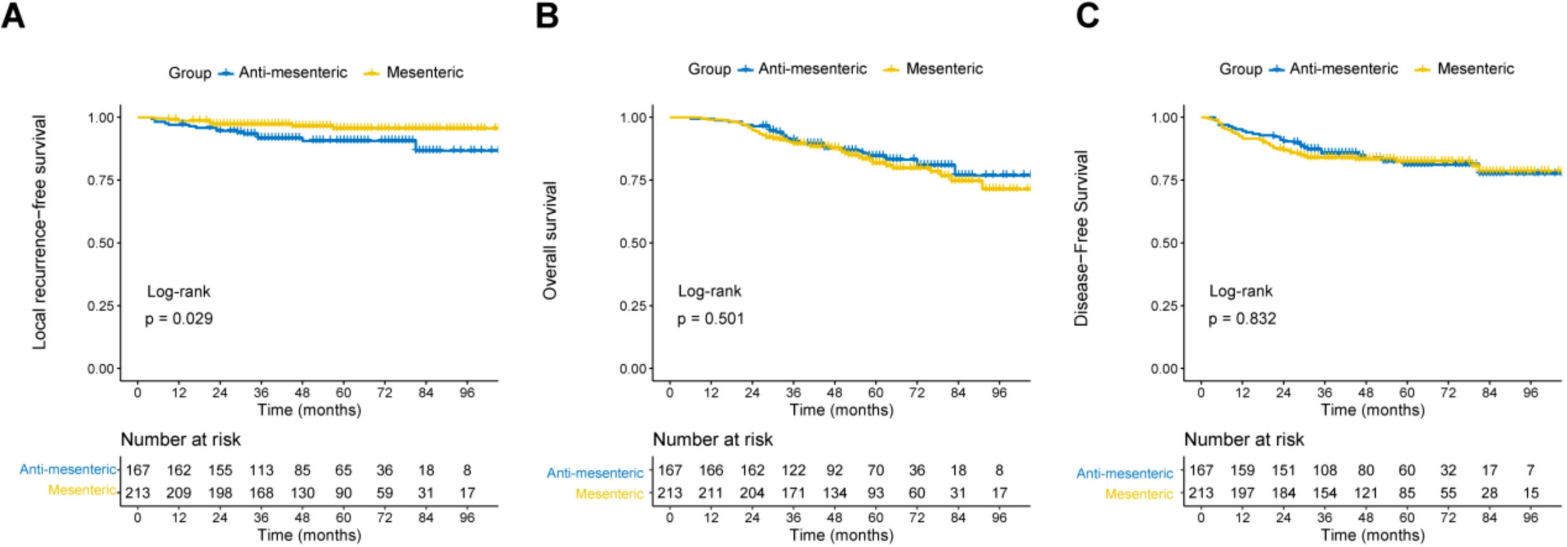
Kaplan-Meier survival curves comparing prognostic outcomes between patients with mesenteric and anti-mesenteric rectal cancer. **(A)** Local recurrence-free survival showed a statistically significant difference between the two groups (log-rank, P = 0.029); **(B)** Overall survival between the mesenteric and anti-mesenteric groups (log-rank, P = 0.501); **(C)** disease-free survival between the two groups (log-rank, P = 0.832).

Kaplan-Meier analysis showed that the 3-year local recurrence-free survival (LRFS) rate was significantly lower in the anti-mesenteric group than in the mesenteric group (91.6% vs. 97.1%, P = 0.029). However, no significant differences were observed between the two groups in overall survival (OS) or disease-free survival (DFS) (**Figure 3**). Univariate analysis revealed that, in addition to axial tumor location, preoperative EMVI, perineural invasion, pathological T stage, and pathological N stage were significantly associated with LRFS (all P < 0.05). DFS was significantly associated with gender, surgical procedure, preoperative MRF, preoperative EMVI, vascular invasion, perineural invasion, pathological T stage, and pathological N stage. OS was significantly associated with age, surgical procedure, preoperative MRF, vascular invasion, pathological T stage, and pathological N stage (**Tables 3**–**5**).

**Table 3:** Univariate and Cox multivariate analysis of factors associated with local recurrence.

Variable	Univariate HR (95% CI)	P	Multivariate HR (95% CI)	P
Age (years)	1.005 (0.967 – 1.044)	0.810	—	—
Gender (Male vs. Female)	1.171 (0.496 – 2.762)	0.719	—	—
BMI (kg/m²)	0.948 (0.840 – 1.071)	0.393	—	—
Surgical procedure (APR vs. LAR)	1.402 (0.520 – 3.780)	0.504	—	—
Longitudinal tumor location (Below vs. Above)	1.271 (0.522 – 3.090)	0.598	—	—
Preoperative MRF (Positive vs. Negative)	1.534 (0.456 – 5.164)	0.490	—	—
Preoperative EMVI (Positive vs. Negative)	2.545 (1.043 – 6.209)	0.040	1.750 (0.691 – 4.435)	0.238
Vascular invasion (Positive vs. Negative)	2.054 (0.887 – 4.758)	0.093	0.906 (0.355 – 2.314)	0.837
Perineural invasion (Positive vs. Negative)	3.283 (1.343 – 8.026)	0.009	1.711 (0.632 – 4.632)	0.291
Axial tumor location (Anti-mesenteric vs. Mesenteric)	2.521 (1.068 – 5.949)	0.035	2.684 (1.126 – 6.398)	0.026
Pathological T stage (pT3/4 vs. pT1/2)	10.388 (1.400 – 77.097)	0.022	1.479 (0.386 – 5.663)	0.567
Pathological N stage (pN+ vs. pN-)	5.073 (1.879 – 13.696)	0.001	3.960 (1.316 – 11.919)	0.014

HR, hazard ratio; CI, confidence interval; BMI, body mass index; MRI, magnetic resonance imaging; MRF, mesorectal fascia; EMVI, extramural vascular invasion; APR, abdominoperineal resection; LAR, low anterior resection.

**Table 4:** Univariate and multivariate analysis for disease-free survival (DFS).

Variable	Univariate HR (95% CI)	P	Multivariate HR (95% CI)	P
Age (years)	0.994 (0.973 – 1.016)	0.604	—	—
Gender (Male vs. Female)	1.844 (1.062 – 3.202)	0.030	1.645 (0.942 – 2.871)	0.080
BMI (kg/m²)	0.986 (0.920 – 1.058)	0.700	—	—
Surgical procedure (APR vs. LAR)	1.830 (1.053 – 3.179)	0.032	1.662 (0.923 – 2.992)	0.090
Longitudinal tumor location (Below vs. Above)	1.195 (0.711 – 2.006)	0.501	—	—
Preoperative MRF (Positive vs. Negative)	2.787 (1.549 – 5.012)	0.001	1.835 (0.953 – 3.534)	0.069
Preoperative EMVI (Positive vs. Negative)	1.951 (1.183 – 3.218)	0.009	1.219 (0.716 – 2.076)	0.465
Vascular invasion (Positive vs. Negative)	2.034 (1.236 – 3.346)	0.005	0.804 (0.443 – 1.459)	0.474
Perineural invasion (Positive vs. Negative)	2.445 (1.471 – 4.066)	0.001	1.413 (0.797 – 2.504)	0.237
Axial tumor location (Anti-mesenteric vs. Mesenteric)	0.949 (0.582 – 1.546)	0.833	—	—
Pathological T stage (pT3/4 vs. pT1/2)	4.839 (2.089 – 11.207)	< 0.001	2.433 (0.981 – 6.036)	0.055
Pathological N stage (pN+ vs. pN-)	4.274 (2.457 – 7.433)	< 0.001	3.088 (1.645 – 5.797)	< 0.001

HR, hazard ratio; CI, confidence interval; BMI, body mass index; MRF, mesorectal fascia; EMVI, extramural vascular invasion; APR, abdominoperineal resection; LAR, low anterior resection.

**Table 5:** Univariate and Cox multivariate analysis of factors associated with overall survival.

Variable	Univariate HR (95% CI)	P	Multivariate HR (95% CI)	P
Age (years)	1.030 (1.005 – 1.055)	0.018	1.043 (1.016 – 1.071)	0.002
Gender (Male vs. Female)	1.404 (0.820 – 2.406)	0.216	—	—
BMI (kg/m²)	0.985 (0.916 – 1.059)	0.678	—	—
Surgical procedure (APR vs. LAR)	2.088 (1.218 – 3.581)	0.007	1.967 (1.105 – 3.502)	0.022
Longitudinal tumor location (Below vs. Above)	0.995 (0.595 – 1.663)	0.983	—	—
Preoperative MRF (Positive vs. Negative)	2.670 (1.450 – 4.916)	0.002	1.630 (0.856 – 3.106)	0.137
Preoperative EMVI (Positive vs. Negative)	1.429 (0.868 – 2.353)	0.160	—	—
Vascular invasion (Positive vs. Negative)	1.907 (1.150 – 3.161)	0.012	0.837 (0.462 – 1.518)	0.559
Perineural invasion (Positive vs. Negative)	1.509 (0.918 – 2.480)	0.104	—	—
Axial tumor location (Anti-mesenteric vs. Mesenteric)	0.840 (0.505 – 1.397)	0.502	—	—
Pathological T stage (pT3/4 vs. pT1/2)	4.649 (2.003 – 10.789)	< 0.001	2.800 (1.151 – 6.811)	0.023
Pathological N stage (pN+ vs. pN-)	4.042 (2.314 – 7.061)	< 0.001	3.683 (1.946 – 6.969)	< 0.001

HR, hazard ratio; CI, confidence interval; BMI, body mass index; MRI, magnetic resonance imaging; MRF, mesorectal fascia; EMVI, extramural vascular invasion; APR, abdominoperineal resection; LAR, low anterior resection.

Furthermore, to evaluate whether the longitudinal tumor location modifies the prognostic impact of the axial tumor location on local recurrence-free survival (LRFS), a stratified subgroup analysis was performed based on the peritoneal reflection (above vs. below). As shown in **Supplementary Figure 1**, the adverse effect of the anti-mesenteric tumor location on LRFS remained consistent across both longitudinal locations (Above peritoneal reflection: HR = 2.113, 95% CI: 0.355–12.576; Below peritoneal reflection: HR = 2.723, 95% CI: 0.978–7.584), and the interaction test confirmed no significant modification effect (P for interaction = 0.764).

### 3.4 Multivariate Cox regression analysis

To adjust for potential confounders, all variables with a P < 0.10 in the univariate analysis (including preoperative EMVI, vascular invasion, perineural invasion, axial tumor location, pathological T stage, and pathological N stage) were entered into a multivariable Cox proportional hazards regression model. As summarized in **Table 3**, for local recurrence-free survival (LRFS), axial tumor location (anti-mesenteric vs. mesenteric) remained an independent risk factor (HR = 2.684, 95% CI: 1.126–6.398, P = 0.026). Additionally, pathological N stage (pN+ vs. pN-) was identified as a strong independent predictor of local recurrence (HR = 3.960, 95% CI: 1.316–11.919, P = 0.014). Regarding disease-free survival (DFS) (**Table 4**), pathological N stage (pN+ vs. pN-) was the only variable confirmed as an independent prognostic factor (HR = 3.088, 95% CI: 1.645–5.797, P < 0.001). For overall survival (OS) (**Table 5**), age (HR = 1.043, 95% CI: 1.016–1.071, P = 0.002), surgical procedure (APR vs. LAR, HR = 1.967, 95% CI: 1.105–3.502, P = 0.022), pathological T stage (pT3/4 vs. pT1/2, HR = 2.800, 95% CI: 1.151–6.811, P = 0.023), and pathological N stage (pN+ vs. pN-, HR = 3.683, 95% CI: 1.946–6.969, P < 0.001) were all confirmed as independent predictors. Axial tumor location was not independently associated with disease-free or overall survival (all P > 0.05).

### 3.5 Sensitivity analysis for local recurrence

Due to the significantly higher proportion of abdominoperineal resections (APRs) in the mesenteric group (20.7% vs. 10.8%, P = 0.014)—a surgical procedure well-recognized as a risk factor for local failure—we performed a targeted sensitivity analysis to test the stability of our primary findings. In the expanded multivariable Cox regression model explicitly incorporating surgical procedure as a covariate (**Supplementary Table 1**), the MRI-defined anti-mesenteric tumor location remained a robust and independent predictor of compromised LRFS. Notably, after adjusting for the surgical procedure, the hazard ratio (HR) for the anti-mesenteric location increased from 2.684 to 2.960 (95% CI: 1.229-7.129, P = 0.015). This demonstrates a classic statistical suppression effect, confirming that the baseline surgical imbalance had initially masked the true magnitude of the independent anatomical risk associated with the anti-mesenteric location. Furthermore, a stratified sensitivity analysis exclusively within the LAR subgroup (n = 318) demonstrated consistent results. Even after completely excluding the APR cases, the anti-mesenteric location remained a strong independent predictor of local recurrence (HR = 4.32, 95% CI: 1.42–13.16, P = 0.010).

## 4 Discussion

Using a standardized MRI-based classification, we found that, compared with mesenteric-side tumors, anti-mesenteric rectal tumors were associated with a higher risk of local recurrence, despite similar baseline clinicopathological features and long-term survival.

Baseline covariate imbalances are frequent challenges in retrospective surgical studies. In our cohort, the mesenteric group featured a significantly higher rate of abdominoperineal resection (APR), a procedure historically associated with compromised local control due to its inherent technical challenges and narrower distal margins (15). However, our sensitivity analysis revealed a compelling clinical phenomenon: despite harboring a higher proportion of these high-risk APR procedures, the mesenteric group still demonstrated superior local control compared with the anti-mesenteric group. When this surgical confounding was rigorously adjusted for in the expanded multivariable Cox model (HR = 2.960, P = 0.015; **Supplementary Table 1**), the independent hazardous effect of the anti-mesenteric location emerged even more prominently. This statistical behavior reinforces the biological hypothesis that the lack of a protective fascial buffer and thick mesorectal fat envelope on the anti-mesenteric side constitutes a fundamental anatomical vulnerability for local recurrence—a risk that persists independently of the baseline surgical approach. Importantly, our stratified analysis limited solely to patients undergoing LAR confirmed that this anatomical vulnerability on the anti-mesenteric side persists independently of the surgical approach.

Furthermore, our updated multivariable survival models highlight the pervasive prognostic impact of nodal involvement, with pathological N stage serving as a consistently strong independent risk factor across local recurrence-free survival (HR = 3.960, P = 0.014), disease-free survival (HR = 3.088, P < 0.001), and overall survival (HR = 3.683, P < 0.001). This aligns with classical staging validations demonstrating that lymph node metastasis remains the most critical determinant of systemic recurrence and long-term survival in rectal cancer (16). Additionally, the multivariable analysis revealed that the abdominoperineal resection (APR) procedure itself is an independent risk factor for worse overall survival (HR = 1.967, P = 0.022). This finding corroborates landmark studies, such as those by Marr et al., which demonstrated that APR is historically associated with compromised survival outcomes and higher rates of margin involvement compared to low anterior resection (15). This underscores the broader systemic impact and historical anatomical challenges associated with the APR approach, further validating our rationale to rigorously adjust for surgical procedures when evaluating the independent risk of the anti-mesenteric tumor location.

Anatomically, the mesorectal side is encased within the mesorectum, providing a clear “holy plane” for TME dissection (17). In contrast, the anti-mesenteric side lacks a well-defined fascial interface and is adjacent to critical pelvic organs, which may lead to incomplete anterior mesorectal excision, narrower resection margins, or intraoperative tumor exposure, thereby increasing recurrence risk. Our findings support this anatomical rationale. Furthermore, as suggested by recent surgical advances, minimally invasive techniques (such as laparoscopy) offer superior pelvic illumination and visual magnification, which theoretically aids in navigating this challenging anterior anatomy (18). Interestingly, despite a high proportion of laparoscopic procedures in our cohort (94.5%), tumors on the anti-mesenteric side still exhibited a higher local recurrence rate. This finding underscores that the inherent anatomical complexity and the lack of a protective fascial envelope on the anti-mesenteric side remain formidable surgical challenges, even with the enhanced visualization provided by modern minimally invasive platforms.

Methodologically, this study introduces a reproducible preoperative MRI-based classification of axial tumor location, which is more objective than postoperative specimen-based assessments. Furthermore, MRI-derived staging showed good correlation with pathological findings.

There are several limitations in this study. First, single center retrospective design may lead to selection bias. Second, to evaluate natural anatomical planes without the confounding effects of radiation-induced tissue fibrosis, we strictly limited our cohort to patients undergoing upfront surgery. We acknowledge that neoadjuvant chemoradiotherapy (nCRT) and, increasingly, total neoadjuvant therapy (TNT) represent the standard-of-care for locally advanced rectal cancers (19–21). Consequently, our findings regarding the prognostic risk of the anti-mesenteric location may be valid exclusively within an upfront surgery population. Extrapolating these anatomical risks to patients who have undergone tumor downstaging via modern neoadjuvant regimens requires caution and warrants future dedicated investigation. Third, the total number of local recurrence events (n=23) remains relatively limited. Although we included all univariable variables with P < 0.10 into the multivariable Cox model to ensure comprehensive adjustment, this approach might inherently carry a risk of model overfitting given the limited number of events. Therefore, these multivariable LRFS findings should be considered somewhat exploratory, and they warrant further validation in larger, multi-center prospective cohorts. This sample size constraint was particularly apparent in our stratified subgroup analysis; although the overall hazard trend for anti-mesenteric tumors remained highly consistent across different longitudinal heights, the statistical power within individual subgroups (above vs. below the peritoneal reflection) was inherently limited due to the divided number of events.

Our findings suggest that the tumor’s axial position may serve as a valuable complementary parameter to the established TNM staging system (22, 23). The presence of tumors on the antimesenteric side during preoperative examination, especially when combined with other high-risk factors like cT3/T4 and MRF invasion, can alert surgeons to potential surgical problems. In this way surgeons can perform more precise dissections, aim for larger margins and discuss local recurrence risks with patients. For these patients, pelvic imaging monitoring post-surgical should also be considered.

Future work should focus on several areas: Validating the results of this study by prospective multicenter cohort, performing subregional analysis of the axial position using radiomics or artificial intelligence, and studying correlation between axial position and specific molecular markers (such as microsatellite instability) to better understand tumor behavior (24–26).

## 5 Conclusion

Axial tumor location is associated with local recurrence risk in rectal cancer, with anti-mesenteric tumors showing higher recurrence rates. While pathological N stage, pathological T stage, age, and surgical procedure remain primary independent prognostic factors for survival outcomes, preoperative MRI assessment of axial location provides valuable supplemental information that may help stratify local recurrence risk, refine surgical planning, and optimize postoperative monitoring.

## Data Availability

The original contributions presented in the study are included in the article/**Supplementary Material**. Further inquiries can be directed to the corresponding authors.

## Supplementary material

Supplementary Table 1 Expanded multivariable Cox regression analysis for local recurrence-free survival (LRFS) incorporating surgical procedure (n=380).

Supplementary Figure 1 Subgroup Analysis of longitudinal tumor location on LRFS.

## References

[1] Bray F Laversanne M Sung H Ferlay J Siegel RL Soerjomataram I . Global cancer statistics 2022: GLOBOCAN estimates of incidence and mortality worldwide for 36 cancers in 185 countries. CA Cancer J Clin. (2024) 74:229–63. doi: 10.3322/caac.21834 38572751

[2] Kapiteijn E Putter H van de Velde CJH . Impact of the introduction and training of total mesorectal excision on recurrence and survival in rectal cancer in The Netherlands. Br J Surg. (2002) 89:1142–9. doi: 10.1046/j.1365-2168.2002.02196.x 12190680

[3] Goedegebuure EP Arico FM Lahaye MJ Maas M Beets GL Peters FP . Defining the tumor location in rectal cancer – Practice variations and impact on treatment decision making. Eur J Surg Oncol. (2025) 51:109700. doi: 10.1016/j.ejso.2025.109700 40106891

[4] Marusch F Koch A Schmidt U Wenisch H Ernst M Manger T . Early postoperative results of surgery for rectal carcinoma as a function of the distance of the tumor from the anal verge: results of a multicenter prospective evaluation. Langenbecks Arch Surg. (2002) 387:94–100. doi: 10.1007/s00423-002-0298-6 12111262

[5] Moszkowicz D Fuks D Gayet B . Surgical anatomy of the rectum. In: Surgical Techniques in Rectal Cancer: Transanal, Laparoscopic and Robotic Approach. Springer Japan, Tokyo (2018). p. 125–46.

[6] Boyle KM Chalmers AG Finan PJ Sagar PM Burke D . Morphology of the mesorectum in patients with primary rectal cancer. Dis Colon Rectum. (2009) 52:1122–9. doi: 10.1007/DCR.0b013e31819ef62f 19581856

[7] Lacy AM Adelsdorfer C . Totally transrectal endoscopic Total Mesorectal Excision (TME). Colorectal Dis. (2011) 13:43–6. doi: 10.1111/j.1463-1318.2011.02781.x 22098517

[8] Liang J-T . Anatomical basis of rectal cancer surgery focused on pelvic fascia. In: Surgical Treatment of Colorectal Cancer: Asian Perspectives on Optimization and Standardization. Singapore: Springer. (2018). p. 37–45. doi: 10.1007/978-981-10-5143-2_4

[9] Fernandes MC Gollub MJ Brown G . The importance of MRI for rectal cancer evaluation. Surg Oncol. (2022) 43:101739. doi: 10.1016/j.suronc.2022.101739 35339339 PMC9464708

[10] Horvat N Petkovska I Gollub MJ . MR imaging of rectal cancer. Radiol Clin. (2018) 56:751–74. doi: 10.1016/j.rcl.2018.04.004 30119772

[11] Dewhurst CE Mortele KJ . Magnetic resonance imaging of rectal cancer. Radiol Clin. (2013) 51:121–31. doi: 10.1016/j.rcl.2012.09.012 23182512

[12] Shihab OC Moran BJ Heald RJ Quirke P Brown G . MRI staging of low rectal cancer. Eur Radiol. (2009) 19:643–50. doi: 10.1007/s00330-008-1184-6 18810451

[13] Amin MB Greene FL Edge SB Compton CC Gershenwald JE Brookland RK . The Eighth Edition AJCC Cancer Staging Manual: Continuing to build a bridge from a population-based to a more “personalized” approach to cancer staging. CA Cancer J Clin. (2017) 67:93–9. doi: 10.3322/caac.21388 28094848

[14] Benson AB Venook AP Al-Hawary MM Azad N Chen Y-J Ciombor KK . Rectal cancer, version 2.2022, NCCN clinical practice guidelines in oncology. J Natl Compr Canc Netw. (2022) 20:1139–67. doi: 10.6004/jnccn.2022.0051 36240850

[15] Marr R Birbeck K Garvican J Macklin CP Tiffin NJ Parsons WJ . The modern abdominoperineal excision: the next challenge after total mesorectal excision. Ann Surg. (2005) 242:74–82. doi: 10.1097/01.sla.0000167926.60908.15 15973104 PMC1357707

[16] Gunderson LL Jessup JM Sargent DJ Greene FL Stewart AK . Revised TN categorization for colon cancer based on national survival outcomes data. J Clin Oncol. (2009) 28:264–71. doi: 10.1200/JCO.2009.24.0952 19949014 PMC2815715

[17] Heald RJ . The ‘Holy plane’ of rectal surgery. J R Soc Med. (1988) 81:503–8. doi: 10.1177/014107688808100904 3184105 PMC1291757

[18] van der Pas MHGM Haglind E Cuesta MA Fürst A Lacy AM Hop WCJ . Laparoscopic versus open surgery for rectal cancer (COLOR II): short-term outcomes of a randomised, phase 3 trial. Lancet Oncol. (2013) 14:210–8. doi: 10.1016/S1470-2045(13)70016-0 23395398

[19] Arnold CR Mangesius J Jäger R Ganswindt U . Neoadjuvant chemoradiotherapy in rectal cancer. Memo. (2020) 13:329–33. doi: 10.1007/s12254-020-00594-0 30311153

[20] Sauer R . Adjuvant and neoadjuvant radiotherapy and concurrent radiochemotherapy for rectal cancer. Pathol Oncol Res. (2002) 8:7–17. doi: 10.1007/BF03033695 11994757

[21] Hodek M Sirák I Ferko A Örhalmi J Hovorková E Hadži Nikolov D . Neoadjuvant chemoradiotherapy of rectal carcinoma. Strahlenther Onkol. (2016) 192:632–40. doi: 10.1007/s00066-016-0988-6 27272661

[22] Aigner F Trieb T Öfner D Margreiter R DeVries A Zbar AP . Anatomical considerations in TNM staging and therapeutical procedures for low rectal cancer. Int J Colorectal Dis. (2007) 22:1339–46. doi: 10.1007/s00384-007-0353-4 17619888

[23] Yang Y Yang Z Lyu Z Ouyang K Wang J Wu D . Pathological-features-modified TNM staging system improves prognostic accuracy for rectal cancer. Dis Colon Rectum. (2024) 67:645–54. doi: 10.1097/DCR.0000000000003034 38147435

[24] Okonkwo A Musunuri S Talamonti M Benson A Small W Stryker SJ . Molecular markers and prediction of response to chemoradiation in rectal cancer. Oncol Rep. (2001) 8:497–500. doi: 10.3892/or.8.3.497 11295069

[25] Edden Y Wexner SD Berho M . The use of molecular markers as a method to predict the response to neoadjuvant therapy for advanced stage rectal adenocarcinoma. Colorectal Dis. (2012) 14:555–61. doi: 10.1111/j.1463-1318.2011.02697.x 21689364

[26] Kim JW Kim YB Choi JJ Koom WS Kim H Kim N-K . Molecular markers predict distant metastases after adjuvant chemoradiation for rectal cancer. Int J Radiat Oncol Biol Phys. (2012) 84:e577–84. doi: 10.1016/j.ijrobp.2012.07.2371 22981710

