# Supplementary material for: Analysis of clinicopathological features and prognosis of mesenteric versus anti-mesenteric rectal cancer: a single-center retrospective cohort study
Source: Front Oncol. 2026 Jul 10;16:1801287. doi: 10.3389/fonc.2026.1801287 (PMC13395617; doi:10.3389/fonc.2026.1801287)
Supplement: Supplementary Table 1 — Expanded multivariable Cox regression analysis for local recurrence-free survival (LRFS) incorporating surgical procedure (n=380). [file Table1.docx]

**Supplementary Material**

**Supplementary Table S1**. Expanded multivariable Cox regression analysis for Local Recurrence-Free Survival (LRFS) incorporating surgical procedure (n=380).

| Variable | Multivariate HR (95% CI) | P |
| --- | --- | --- |
| Surgical procedure (APR vs. LAR) | 1.707（0.620 – 4.702） | 0.301 |
| Perineural invasion (Positive vs. Negative) | 1.762 (0.691 – 4.499) | 0.236 |
| Axial tumor location (Anti-mesenteric vs. Mesenteric) | 2.960（1.229 – 7.129） | 0.015 |
| Pathological N stage (pN+ vs. pN-) | 4.528 (1.588 – 12.910) | 0.005 |
