# Supplementary material for: Analysis of clinicopathological features and prognosis of mesenteric versus anti-mesenteric rectal cancer: a single-center retrospective cohort study
Source: Front Oncol. 2026 Jul 10;16:1801287. doi: 10.3389/fonc.2026.1801287 (PMC13395617; doi:10.3389/fonc.2026.1801287)

**Supplementary Material**

**Supplementary Figure S1.** Subgroup Analysis of longitudinal tumor location on LRFS


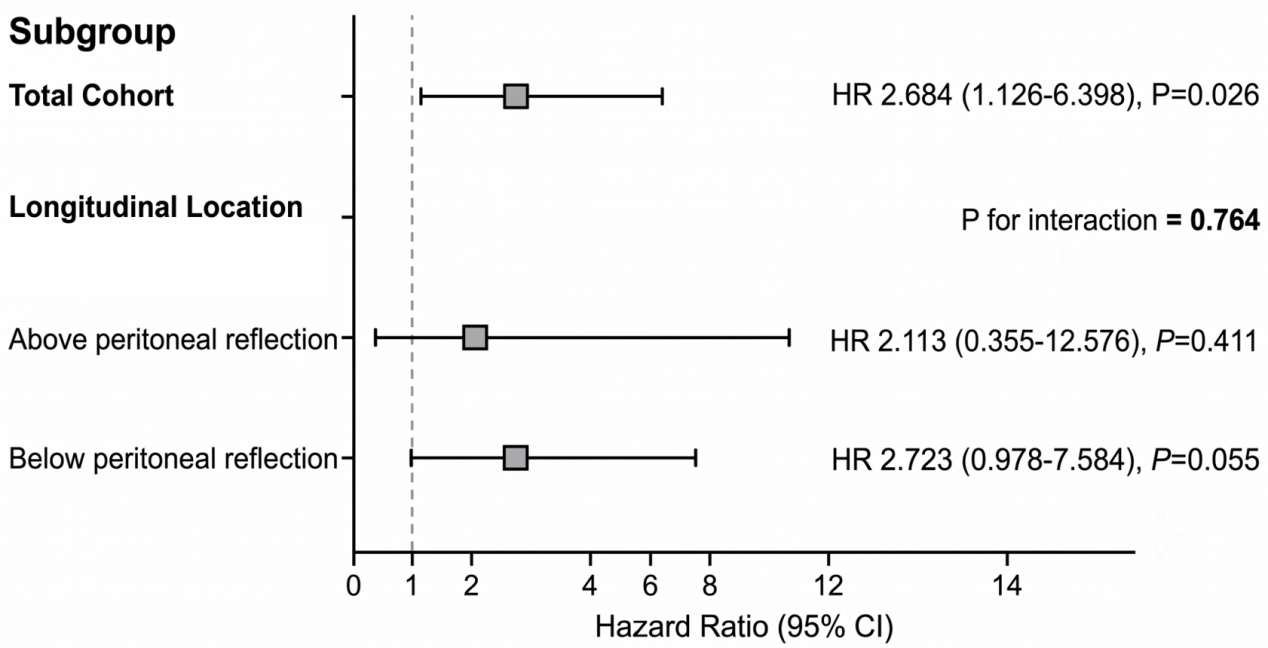

Supplement: Supplementary Figure 1 — Subgroup Analysis of longitudinal tumor location on LRFS. [file Supplementaryfile1.docx]
